# Land- and water-based aerobic exercise program on health-related outcomes in breast cancer survivors (WaterMama): study protocol for a randomized clinical trial

**DOI:** 10.1186/s13063-024-08389-y

**Published:** 2024-08-13

**Authors:** Cristine Lima Alberton, Luana Siqueira Andrade, Bruno Ezequiel Botelho Xavier, Victor Hugo Guesser Pinheiro, Antonio Ignacio Cuesta-Vargas, Stephanie Santana Pinto

**Affiliations:** 1 Escola Superior de Educação Física e Fisioterapia, Universidade Federal de Pelotas, Pelotas, Brazil; 2 Facultad de Ciencias de La Salud, Universidad de Málaga, Málaga, Spain

**Keywords:** Physical exercise, Breast neoplasm, Physical activity, Aquatic exercise, Cancer-related fatigue, Physical fitness, Mental health, Cognitive function, Pain, Quality of life

## Abstract

**Background:**

Breast cancer is a prevalent form of cancer among women worldwide, often accompanied by physical and psychological side effects due to the disease and the treatment’s aggressiveness. Regular physical exercise has emerged as a non-pharmacological approach to improve the quality of life of breast cancer survivors. We herein report the protocol of the WaterMama Study, which aims to evaluate the effects of land- or water-based aerobic exercise programs, compared to a health education program, on cancer-related fatigue and other health-related outcomes in breast cancer survivors.

**Methods:**

The WaterMama trial is a randomized, single-blinded, three-arm, parallel, superiority trial. We aim to recruit 48 women ≥ 18 years of age who have completed primary treatment for stage I–III breast cancer. Participants are randomly allocated in a 1:1:1 ratio to 12-week interventions of aerobic exercise training programs either in the aquatic or land environment (two weekly 45-min sessions) plus health education (a weekly 45-min session), or an active-control group receiving health education alone (a weekly 45-min session). The primary outcome is cancer-related fatigue, and the secondary outcomes include cardiorespiratory fitness, muscular performance, muscle morphology, functional capacity, mental health, cognitive function, pain, and quality of life. Outcomes assessments are conducted before and after the 12-week intervention period. The analysis plan will employ an intention-to-treat approach and per protocol criteria.

**Discussion:**

Our conceptual hypothesis is that both aerobic exercise programs will positively impact primary and secondary outcomes compared to the health education group alone. Additionally, due to its multi-component nature, we expect the aquatic exercise program promote more significant effects than the land exercise program on cancer-related fatigue, muscular outcomes, and pain.

**Trial registration:**

The study was prospectively registered at ClinicalTrials.gov NCT05520515. Registered on August 26, 2022. https://clinicaltrials.gov/ct2/show/NCT05520515

## Administrative information

Note: the numbers in curly brackets in this protocol refer to SPIRIT checklist item numbers. The order of the items has been modified to group similar items (see http://www.equator-network.org/reporting-guidelines/spirit-2013-statement-defining-standard-protocol-items-for-clinical-trials/).

**Table:** 

Title {1}	Land- and water-based aerobic exercise program on health-related outcomes in breast cancer survivors (WaterMama): study protocol for a randomized clinical trial
Trial registration {2a and 2b}	Water- and land-based aerobic training in breast cancer survivors (WaterMama). NCT05520515
Protocol version {3}	Initial released at ClinicalTrials.gov: August 23, 2022 Last update at ClinicalTrials.gov: February 02, 2023
Funding {4}	The WaterMama Study has been funded by CNPq (Conselho Nacional de Desenvolvimento Científico e Tecnológico), Brazil, Grant number 431288/2018–6. In addition, involved personnel have been funded by institutions listed below: CNPq (Conselho Nacional de Desenvolvimento Científico e Tecnológico), Brazil, Grant number 315430/2021–4 (CLA) CAPES (Coordenação de Aperfeiçoamento de Pessoal de Nível Superior), Brazil, Finance Code 001 (VHGP) FAPERGS (Fundação de Amparo à Pesquisa do Estado do Rio Grande do Sul), Brazil, Grant number 23/2551–00011846-2 (LSA)
Author details {5a}	CLA: Escola Superior de Educação Física e Fisioterapia, Universidade Federal de Pelotas, Pelotas, Brazil LSA: Escola Superior de Educação Física e Fisioterapia, Universidade Federal de Pelotas, Pelotas, Brazil BEBX: Escola Superior de Educação Física e Fisioterapia, Universidade Federal de Pelotas, Pelotas, Brazil VHGP: Escola Superior de Educação Física e Fisioterapia, Universidade Federal de Pelotas, Pelotas, Brazil AICV: Facultad de Ciencias de la Salud, Universidad de Málaga, Málaga, Spain SSP: Escola Superior de Educação Física e Fisioterapia, Universidade Federal de Pelotas, Pelotas, Brazil
Name and contact information for the trial sponsor {5b}	Universidade Federal de Pelotas R. Gomes Carneiro, 01—Balsa, Pelotas—RS, 96,010–610 + 55(53)3284–4006 https://portal.ufpel.edu.br/
Role of sponsor {5c}	The sponsor (Federal University of Pelotas, Brazil) played no part in study design; collection, management, analysis, and interpretation of data; writing of the report; and the decision to submit the report for publication.

## Introduction

### Background and rationale {6a}

Breast cancer stands as the most prevalent cancer type among women worldwide. In 2022, it accounted for approximately 2.3 million new diagnoses, representing 23.8% of all new cases in women and the most frequent cause of death (666,103 deaths) across all age groups [1]. In Brazil, excluding non-melanoma skin tumors, it is the most incident cancer among women across all regions of the country. Projections for 2023 to 2025 estimate 73,610 new breast cancer cases in Brazil, corresponding to a risk of 66.54 new cases per 100,000 women [2].

Scientific evidence has confirmed that cancer treatment enhances survival rates [3]. However, survival is often accompanied by physical and psychological side effects caused by both the disease and the toxicity of the treatment. Persistent symptoms include exacerbated cancer-related fatigue, impaired quality of life, increased depressive and anxiety symptoms, and compromised ability to perform daily activities [4–10]. Notably, cancer-related fatigue is reported as one of the most substantial challenges faced by breast cancer survivors [11, 12], with a consequent negative impact on the health-related quality of life, underscoring the paramount importance of investigating this outcome within this population.

In this context, physical exercise is considered a non-pharmacological approach to improving health-related outcomes for cancer survivors [13]. Meta-analyses of several controlled intervention studies have demonstrated that physical exercise programs can improve cancer-related fatigue, physical fitness, and mental health in this population [11, 12, 14]. While evidence supporting the positive effects of exercise programs during and after breast cancer treatment has predominantly focused on land-based exercise programs (i.e., walking/running, cycling, resistance training) [15], there is growing interest in the potential benefits of water-based exercise programs for this population [16–19]. While original evidence is increasing in this area [20–29], there is a notable lack of studies comparing the effects of exercise programs in aquatic and land environments for breast cancer survivors [24, 26].

An important characteristic of the aquatic environment is the lower apparent weight resulting from water immersion, corresponding to around 20 to 30% of the total body weight on land in women with different age ranges and body compositions [30, 31]. This substantially reduced load to be supported during water-based exercises facilitates exercise performance and reduces osteoarticular impact. Additionally, the physical properties of the aquatic environment provide analgesic effects in a pleasant setting [20], promoting well-being and favoring adherence to exercise programs. The large sensory flow in water may be responsible for reduced pain perception, with pain modulation affected by factors such as water temperature and turbulence (therapeutic effect of hydromassage) [32]. This aspect may be particularly beneficial for breast cancer survivors undergoing hormonal therapy, as joint pain and other musculoskeletal disorders may arise as possible adverse effects [33].

Moreover, water-based exercises may offer advantages due to their multi-component nature. A program involving aerobic exercises performed in water can yield improvements not only in cardiorespiratory parameters but also in flexibility, balance, and muscle strength, thickness, and quality [34–36]. These characteristics may be attributed to the water’s physical properties, such as drag force and buoyancy, which provide a multidirectional resistance and a higher instability than the land environment during exercise performance [37]. Although such outcomes were primarily investigated in older populations, it is plausible that these findings may be applicable to breast cancer survivors. Considering the physical and psychological impairments in breast cancer survivors, it is pertinent to investigate if exercise programs performed in the aquatic environment may potentiate health-related benefits.

### Objectives {7}

We aim to analyze the effects of 12 weeks of land- or water-based exercise programs compared with a health education program alone on cancer-related fatigue, cardiorespiratory fitness, muscular performance, muscle morphology, functional performance, mental health, cognitive function, pain, and quality of life in breast cancer survivors. Herein, we report the protocol of the WaterMama Study, a superiority randomized clinical trial with three parallel groups, using cancer-related fatigue as the primary outcome. Based on the intervention design and outcomes of interest, we hypothesize that both training programs will significantly improve the investigated outcomes compared to the health education group alone. Additionally, we expect the water-based exercise program to have a more significant positive impact on cancer-related fatigue and physical parameters due to its multi-component nature.

### Trial design {8}

The WaterMama trial is a randomized clinical trial employing a 1:1:1 allocation ratio. It was designed as a superiority trial with three parallel groups, controlled by active intervention, and blinded to outcome assessors and data analysts.

## Methods: participants, interventions, and outcomes

### Study setting {9}

This trial is being conducted at the Escola Superior de Educação Física e Fisioterapia (ESEF) of the Universidade Federal de Pelotas (UFPel), Brazil.

### Eligibility criteria {10}

The inclusion and exclusion criteria for participants are outlined as follows.

#### Inclusion criteria

1. Women diagnosed with stage I–III breast cancer
2. Aged 18 years or older
3. Have completed primary treatment for breast cancer (including surgery, chemotherapy, and/or radiotherapy) within the past 24 months before the start of the intervention, regardless of hormonal treatment status
4. Willingness to participate in either intervention group and absence of fear of the aquatic environment

#### Exclusion criteria

1. Active metastatic or locoregional disease
2. Presence of major psychiatric or cognitive disorders
3. Severe nausea, anorexia, or any other condition that impedes participation in the exercise
4. Regular engagement in exercise within the last 3 months

### Who will take informed consent? {26a}

During the recruitment process, potential participants are invited to visit the Laboratório de Avaliação Neuromuscular (LabNeuro) at ESEF/UFPel after the initial phone contact and screening to verify eligibility criteria. During this visit, one of the responsible researchers (BEBX or VHGP) provides detailed information about the research procedures, including potential risks and benefits, and addresses any possible questions. Finally, participants are asked to sign an informed consent form if they agree to participate.

### Additional consent provisions for collection and use of participant data and biological specimens {26b}

Not applicable. All necessary consent for data collection is included in the primary informed consent, and no biological specimens will be collected.

## Interventions

### Explanation for the choice of comparators {6b}

Participants are allocated after randomization into one of three groups: a water-based exercise training group, a land-based exercise training group, or a health education active control group, all lasting for 12 weeks. The choice of a land-based training group is a crucial comparator in this study. Comparing the outcomes of an aquatic program with those of a land program allows a comprehensive assessment of the potential additional benefits conferred by the aquatic environment. A land-based comparator will allow discerning whether the unique physical properties of water, such as drag force and buoyancy, offer advantages beyond those achievable through traditional land-based exercises. In addition, the decision to incorporate a health education comparator was motivated by the need to have a control group that does not engage in exercise but still receives some form of intervention. By selecting health education as the active comparator, we acknowledge the importance of not only physical exercise but also health education and awareness in promoting overall well-being. To ensure that the effect of the health education intervention did not make this group substantially different from the others, activities from the health education group were similarly integrated into the other intervention groups.

### Intervention description {11a}

#### Active control group: health education program

The health education sessions take place at the ESEF/UFPel. Participants from the active control group engage in an educational program consisting of 45-min lectures once a week. These lectures are led by a qualified health professional following a structured content script using both expository and interactive approaches. The topics covered include breast cancer symptoms and therapeutic management. Participants also receive a customized booklet (available in press and digital formats) at the study’s onset containing the lecture topics presented in Table 1.

**Table 1:** Topics covered in the health education intervention

Topics in health education for hypertension
1. Body image
2. Arm, breast, and vasomotor symptoms
3. Quality of life
4. Eating habits
5. Cancer-related fatigue
6. Integrative and complementary practices
7. Cognitive function
8. Quality of sleep
9. Depressive and anxiety symptoms
10. Pain and arthralgia
11. Sexuality
12. Physical activity and exercise

#### Exercise intervention groups: water- and land-based exercise programs

Participants of both exercise intervention groups undertake a 12-week exercise program with two weekly sessions on non-consecutive days, regardless of the exercise environment. Each session lasts 45 min and comprises a 5-min warm-up, 35 min of aerobic exercises, and a 5-min stretching routine throughout the intervention. In addition, both exercise intervention groups participate in the same educational program as the health education group, receiving lectures separately without interacting with the other groups.

Training intensity is prescribed using Borg’s 6–20 rating of perceived exertion (RPE) scale. This method was chosen due to the physiological changes induced by immersion, such as heart rate (HR) alterations, requiring specific maximal tests in each environment to determine the HR training target [38]. RPE provides a valid tool for intensity control, exhibiting similar indexes at submaximal and maximal intensities between environments [39]. Studies employing RPE to prescribe water-based aerobic training have revealed positive effects on cardiorespiratory, neuromuscular, and functional parameters in various populations [35, 36].

The exercise program’s periodization was adapted from those proposed by Andrade et al. [35], as detailed in Table 2. This strategy periodization is based on interval training, alternating between high-intensity effort stimulus and low-intensity active recovery phases. To ensure controlled progression of external load throughout the exercise interventions in aquatic and land environments, the number of repetitions and the distance covered during the first minute of the intensity stimulus in the first and seventh sets were individually recorded at the last session of each mesocycle (sessions 6, 12, 18, and 24).

**Table 2:** 12-week aerobic exercises duration

Weeks	Sets	Intensity	Duration
1–3	7	4 min RPE 13 + 1 min RPE 11	35 min
4–6	7	4 min RPE 14 + 1 min RPE 11	35 min
7–9	7	4 min RPE 15 + 1 min RPE 11	35 min
10–12	7	4 min RPE 16 + 1 min RPE 11	35 min

*RPE* rating of perceived exertion

##### Water-based exercise program

The intervention takes place at the thermal pool of the Brilhante Club in Pelotas City. The pool water temperature is maintained between 30 and 32 °C, with participants immersed to a depth level between the xiphoid process and shoulders. The water-based exercise session comprises the following exercises sequence: stationary running, frontal kick, cross-country skiing, and butt kick during the stimulus, and jumping jacks during the active recovery. These exercises are commonly used, featuring stationary execution, and offer safe osteoarticular impact loads suitable for women of different age and body composition ranges [30, 31]. The exercise sessions are conducted in small groups, with a maximum of eight participants, supervised by two experienced instructors, one outside and one inside the pool. An RPE scale (banner 0.60 × 0.90 m) is fixed outside the pool in front of the participants.

##### Land-based exercise program

The intervention takes place at the multi-sport gym of the ESEF/UFPel. The land-based exercise session comprises walking/running on the court. The exercise sessions are conducted in small groups, with a maximum of eight participants, supervised by two experienced instructors, one positioned in each corner of the court. An RPE scale (banner 0.60 × 0.90 m) is fixed at the corner corresponding to the starting point.

### Criteria for discontinuing or modifying allocated interventions {11b}

Participants may be discontinued from the study if they withdraw their consent, lose interest, or are unwilling to continue. Participation will be interrupted for individuals allocated to any group in case of safety concerns, such as medical advice, complications arising from the disease, or a severe health event that precludes attendance at intervention sessions.

### Strategies to improve adherence to interventions {11c}

Participants allocated to the active control group receive text messages 2 days before the health education session to reinforce the date and time of the meeting. The exercise training groups receive messages at the beginning of each week, reinforcing the interventions’ date, time, and location. We use phone calls or WhatsApp messages to inquire about adverse events in cases where a participant misses a session from any intervention group. The call schedule is interrupted for participants who declare their withdrawal from the study.

### Relevant concomitant care permitted or prohibited during the trial {11d}

Participants were instructed not to engage in any other type of activity involving exercise during the study period. Participants were also advised to maintain usual care treatment for breast cancer, if applicable, or another coexisting disease.

### Provisions for post-trial care {30}

All participants interested in continuing physical exercises practice after the study’s conclusion will be invited to join the Exercise Research in Cancer (ERICA) Extension Project conducted in the ESEF/UFPel. This project offers a free supervised physical exercise program consisting of two sessions of 1-h per week, including aerobic and resistance exercises. This program is available to all breast cancer survivors who participated in studies associated with our laboratory.

### Outcomes {12}

The primary outcome of the study is cancer-related fatigue, measured before and after 12 weeks of intervention, using the Piper Fatigue Scale (PFS). Data are presented as group means at baseline and post-intervention time points in the assessment. Cancer-related fatigue was chosen as the primary outcome because it is one of the main complaints among breast cancer survivors [11, 12].

A set of clinically relevant secondary outcomes for breast cancer survivors was established, considering the side effects of cancer and its treatment. The secondary outcomes are measured before and after 12 weeks of intervention, presented as group means at baseline and post-intervention time points in the measurements, and include:

- Cardiorespiratory fitness: determined by peak oxygen consumption (VO_2peak_) and ventilatory thresholds (i.e., oxygen uptake in the first ventilatory threshold—VO_2VT1_ and oxygen uptake in the second ventilatory threshold—VO_2VT2_) obtained by an incremental test on a treadmill.
- Muscular performance: determined by the maximum dynamic muscular strength and dynamic muscular endurance of the knee extensors.
- Muscle morphology: determined by muscle thickness and muscle quality of the quadriceps femoris, measured from images obtained by B-mode ultrasonography.
- Functional performance: determined by functional tests performed according to the procedures proposed by Rikli and Jones [40].
- Mental health: measured by depressive and anxiety symptoms using the Hospital Anxiety and Depression Scale (HADS).
- Cognitive function: determined by aspects of perceived cognitive function measured using the Functional Assessment of Cancer Therapy—Cognitive Function—Version 3 (FACT-Cog-v3) questionnaire and aspects of objective cognitive function measured by the Trail Making Test (TMT) and the Controlled Oral Word Association Test (COWAT).
- Pain: measured by the Brief Pain Inventory (BPI).
- Quality of life: measured by the Functional Assessment of Breast Cancer Therapy (FACT-B) instrument.

### Participant timeline {13}

The schedule of trial enrollment, interventions, and assessments are presented in Table 3.

**Table 3:** Time scheme for study conduction

	Study period							
	Enrollment	Baseline measures		Allocation	Post-allocation		Close out	
Timepoint	T-1	T0^a^		T1	T2	T3^b^	T4^ab^	
Timepoint description		Evaluation visit 1	Evaluation visit 2		Intervention start	Intervention end	Final evaluation visit 1	Final evaluation visit 2
Enrollment								
Eligibility screening	✕							
Informed consent	✕							
Allocation				✕				
Interventions								
Aquatic training program plus health education					✕	✕		
Land training program plus health education					✕	✕		
Health education program					✕	✕		
Assessments								
Primary outcome								
Cancer-related fatigue questionnaire		✕					✕	
Secondary outcomes								
Cardiorespiratory fitness			✕					✕
Maximal dynamic strength		✕					✕	
Dynamic muscular endurance		✕					✕	
Functional tests		✕					✕	
Muscle thickness and muscle quality			✕					✕
Depression and anxiety scale		✕					✕	
Cognitive function questionnaire and tests		✕	✕				✕	✕
Pain inventory			✕					✕
Quality of life questionnaire		✕					✕	
Additional measurements								
Anthropometric measurements			✕					✕
Physical activity levels			✕					✕
Food consumption			✕					✕
Office blood pressure			✕					✕
Follow-up questionnaire								✕

^a^period will be no longer than 2 weeks

^b^time between t3 and t4 will be no longer than 2 weeks

### Sample size {14}

The sample calculation was conducted using the GPower version 3.1 program, adopting a significance level of *α* = 0.05 and 90% power. Data for the calculation were derived from the study results by Kessels et al. [12] for the primary outcome of cancer-related fatigue (effect size 0.605). It resulted in a total sample size of 39 subjects. Additionally, nine individuals (approximately 20%) will be included in the study to account for potential sample losses, totaling 48 participants randomized into the three groups.

### Recruitment {15}

The recruitment period began in January 2023 and is expected to conclude by August 2024. Upon authorization from the Head of the Oncology Service at Hospital Escola/UFPel and Santa Casa de Misericórdia de Pelotas, lists of service users’ telephone numbers are compiled from the corresponding medical records departments. These individuals are then contacted by phone and WhatsApp messages and invited to participate in the study. Additionally, the study is promoted through announcements in local or regional newspapers and on social media, including the contact information for interested individuals. During the phone contact, individuals receive comprehensive information about the study’s purpose and undergo a screening to determine their eligibility based on inclusion criteria.

The potential eligible participants are then invited to visit the LabNeuro at ESEF/UFPel for an individual interview with one of the responsible researchers (BEBX or VHGP). During this initial visit, detailed information about the research procedures, including questions regarding the exclusion criteria, is provided. Eligible participants are fully informed about all procedures, including potential risks and benefits, and are asked to sign an informed consent form if they agree to participate.

## Assignment of interventions: allocation

### Sequence generation {16a}

The randomization sequence is generated on the website www.random.org, with a 1:1:1 proportion and stratification by endocrine therapy (aromatase inhibitors, selective estrogen receptor modulators, and no endocrine therapy). The sequence is based on randomly sized blocks that are not disclosed to ensure concealment.

### Concealment mechanism {16b}

Upon enrollment in the study, each participant is assigned an internal identifying number (ID) that is used for the allocation sequence. A blinded researcher implements the allocation after the baseline evaluation conclusion by accessing the randomization list based on the participants’ ID. Participants are allocated into one of the three groups and informed about their intervention (water-based exercise intervention group, land-based exercise intervention group, or health education active control group) via telephone or message.

### Implementation {16c}

LSA is the researcher responsible for generating the allocation sequence, CLA is the researcher responsible for enrolling participants, and VHGP is the researcher responsible for assigning participants to interventions.

## Assignment of interventions: blinding

### Who will be blinded {17a}

Blinding is implemented for outcome assessors and data analysts responsible for evaluating both primary and secondary outcomes. However, due to the inherent nature of the interventions, neither the staff conducting the exercise or health education sessions, nor the participants are blinded. Participants are instructed to refrain from disclosing their assigned group and discussing their interventions during outcomes assessments to ensure the assessor masking.

### Procedure for unblinding if needed {17b}

In the case of unintentional unblinding for any reason, researchers involved are required to notify the coordinator. This information is then documented for internal control purposes.

## Data collection and management

### Plans for assessment and collection of outcomes {18a}

On the initial visit, after signing the consent form, each participant completes a questionnaire covering their clinical and sociodemographic characteristics. During this visit, familiarization procedures are also performed with the knee extension equipment, treadmill, and RPE scale to mitigate potential learning biases and ensure participants’ proficiency in performing the tests before formal data collection.

The study outcomes are assessed at two time points: baseline (weeks 0) and post-intervention (week 13). All randomized participants undergo outcome assessments, irrespective of their attendance or completion status. Participants who withdraw from the study at any time after randomization are still invited to complete the final evaluations (week 13), ensuring data inclusion in the intention-to-treat analysis. Measurements are performed in two separate visits, apart at 48-h interval to avoid interference among them, according to the sequence:

- Visit 1: quality of life questionnaire, cancer-related fatigue questionnaire, perceived cognitive function questionnaire, mental health questionnaire, maximal dynamical strength of knee extensors, dynamic muscular endurance of knee extensors, and functional tests
- Visit 2: quality and thickness of quadriceps, objective cognitive function instruments, pain questionnaire, physical activity levels questionnaire, anthropometric measurements, office blood pressure, cardiorespiratory fitness test and food frequency questionnaire

Participants are instructed to avoid intense physical activity 72 h before the first evaluation session (week 0). Post-intervention evaluations take place 72 h after the last training session. In addition, the follow-up questionnaire is applied on the last visit of the final evaluations.

The same investigator applies each test at baseline and post-intervention time points to ensure consistency. Outcome assessors are trained, and standardized procedures are performed for each assessor during assessments. In addition, the same investigator applies the questionnaires individually at baseline and post-intervention time points in a reserved room.

#### Measurement of the primary outcome

##### Cancer-related fatigue

Cancer-related fatigue is measured using the PFS, a valid and reliable instrument for assessing fatigue in Brazilian cancer patients [41]. The PFS consists of 22 numerical items that assess the fatigue experienced by patients, using a 0–10 numeric scale. It measures four dimensions of subjective fatigue: behavioral/severity, affective meaning, sensory, and cognitive/mood. The total fatigue score is calculated by averaging the four subscale scores.

#### Measurements of secondary outcomes

##### Cardiorespiratory fitness

Cardiorespiratory fitness is determined by VO_2peak_ and ventilatory thresholds (i.e., VO_2VT1_ and VO_2VT2_) obtained by an incremental test on a treadmill (KIKOS, São Paulo—São Paulo, Brazil). First, the participants are kept seated at rest for 5 min in a calm environment to take HR measurements at rest using a heart rate monitor (H10, Polar, Kempele, Finland). Warming up is carried out for 3 min with a gradual increase in speed until reaching 3 km·h^−1^, and then the test starts at 3 km·h^−1^ with sequential increments of 0.5 km·h^−1^ every minute and a 1% increase in grade every 2 min until maximum effort. The test is finished when the participant can no longer exercise at a given workload, indicating exhaustion. All tests are supervised by a trained exercise physiologist and a physician.

Oxygen uptake is recorded during the incremental protocol using a mixing-box-type portable gas analyzer (VO2000, MedGraphics; Ann Arbor, USA) to determine participants’ VO_2peak_, VO_2VT1_, and VO_2VT2_. Data are acquired as an average of three breaths using the Aerograph software (MedGraphics; Ann Arbor, USA). Heart rate (HR) is obtained every 15 s using a heart rate monitor (FT1, Polar, Kempele, Finland), and participants’ RPE is assessed at the end of each stage with the Borg’s RPE 6–20 scale.

Tests are considered valid when at least two of the following criteria are met at the end of the test: maximal respiratory exchange rate ≥ 1.1, maximal estimated heart rate ≥ 220 bpm less age, and a maximal RPE ≥ 18 [42]. The individual VO_2peak_ is determined as the higher 15 s mean oxygen uptake value in the last test stage. The VT1 and VT2 are determined based on the ventilation by test stage plot and confirmed by the ventilatory equivalent of oxygen (VE/VO_2_) and carbon dioxide (VE/VCO_2_), respectively [43]. Two experienced physiologists independently detect thresholds by visual inspection while blinded to the participants’ experimental group. When there is no agreement among them, the opinion of a third physiologist is requested.

##### Muscular performance

The maximum dynamic muscle strength of knee extensors is measured the one-repetition maximum (1RM) test in a knee extension equipment (Sportmania Fitness, Novo Hamburgo, RS, Brazil). The 1RM value is considered the greatest load that the participant could lift for one complete repetition (i.e., concentric and eccentric phase) following a predetermined cadence (i.e., approximately 2 s per phase) controlled by a digital app (Metronome). The 1RM of each participant is determined within five attempts, and at least 3 min of rest interval was given between trials. A new load was estimated following Lombardi’s coefficient [44] for the subsequent trial when the participant could perform more than one complete repetition. The test is rescheduled if the value of 1RM is not determined between the five attempts. According to a previous study from our laboratory [35], the range of motion is being individualized for each participant and controlled by a range of motion custom-build device.

The same knee extension equipment is used to assess dynamic muscular endurance. Participants perform the maximal number of bilateral knee extension repetitions with 60% of individual 1RM load. The test cadence (2 s for each contraction phase) and range of motion are the same for the 1RM test. The post-intervention assessment is performed using the same absolute load employed at baseline (i.e., 60% of baseline 1RM).

##### Muscle morphology

Muscle thickness and muscle quality of the quadriceps femoris are measured from images obtained by B-mode ultrasonography (Toshiba—Tosbee/SSA-240A, Japan). Initially, the participants rest for 5 min in a supine position with legs extended and relaxed to stabilize the displacement of body fluids. Then, transversal images of the four portions of the quadriceps femoris are recorded with a 7.5-MHz linear array probe. Images of the vastus lateralis (VL), rectus femoris (RF), and vastus intermedius (VI) muscles are obtained at the midpoint between the anterosuperior iliac spine and the upper edge of the patella, whereas the vastus medialis (VM) is assessed at 30% of the distance between the lateral condyle and the greater trochanter of the femur, based on a previous study [35]. To ensure a similar probe position in subsequent tests, the assessment site of each muscle is marked on transparent paper and used for probe repositioning.

All images will be analyzed using the ImageJ software (National Institutes of Health, USA, version 1.37). The muscle thickness will be assessed as the distance from each muscle’s superior and inferior muscle aponeurosis [45]. Overall quadriceps femoris muscle thickness will be calculated as the sum of each muscle thickness (i.e., RF + VL + VM + VI). Muscle quality will be determined by the echo intensity values, which will be calculated from gray-scale analysis using the standard histogram function in ImageJ (National Institute of Health, USA, version 1.37). A region of interest will be selected in each muscle, including as much of the muscle as possible while avoiding surrounding fascia, and the echo intensity value within the region of interest will be calculated and expressed in values between 0 and 255 (0 = black; 255 = white [46]). The echo intensity of the quadriceps femoris will be calculated as the mean of echo intensity values of the four individual quadriceps femoris muscles ((RF + VL + VM + VI)/4).

##### Functional performance

Functional tests are performed according to the procedures proposed by Rikli and Jones [40].

The Arm Curl test is performed to measure the strength of the upper limbs. Starting at full elbow extension and holding a 2-kg dumbbell in each hand, participants are instructed to perform the maximal number of elbow crunches over the full range of motion for 30 s. The test is performed with both upper limbs.

The 30-s Chair-Stand test is performed to measure the strength of the lower limbs. Participants are instructed to sit and stand up from a chair 43 cm high from the seat, without the aid of the upper limbs, as many times as possible for 30 s.

The 8-ft Up-and-Go test is performed to measure agility and dynamic balance. Participants are instructed to get up from the chair (43 cm), turn around a marker that will be 2.44 m, and return to the starting position. The shortest time of two attempts will be considered as a result.

The Chair Sit-and-Reach test is performed to measure the flexibility of the lower limbs. Participants sit on the front edge of a chair and extend one leg straight out in front of the hip, with the foot flexed and the heel resting on the floor (the other leg is bent, foot flat on the floor). The object is to reach as far forward as possible toward (or past) the toes. The investigator uses a ruler to note the cm left to reach the toe (negative score) or the cm that went past the toe (positive score).

The Back Scratch test is performed to measure the flexibility of the upper limbs. Participants are instructed to try to touch the middle fingers of both hands together behind the back. The investigator uses a ruler noting the cm left to reach the middle fingers (negative score) or the cm that the middle fingers overlapped (positive score).

The 6-min Walk test is performed to measure aerobic fitness. The course proposed in the original test is 45.72 m rectangular. The course will be adapted for a straight line of 30 m in length, demarcated with cones every 3 m. Participants are instructed to walk for 6-min in a flat 30 m course, where the total distance walked “as fast as possible” is assessed.

##### Mental health

HADS was developed by Zigmond and Snaith [47] to evaluate depressive and anxiety symptoms. The instrument was translated and validated for the Brazilian population [48]. HADS consists of 14 items, with seven items forming the anxiety subscale and the other seven forming the depression subscale. It allows for the assessment of symptoms experienced in the previous week. Each item has four response options, ranging from 0 to 3, with each subscale having a maximum score of 21 points.

##### Cognitive function

Perceived cognitive function is measured using the FACT-Cog-v3, an instrument specifically developed for cancer patients [49]. It consists of 37 items organized into four sections: perceived cognitive impairments, comments from others, perceived cognitive abilities, and impact on quality of life. Each item has five response options, and the recall period covers the past 7 days.

Objective cognitive function is measured using the TMT and the COWAT. The TMT assesses domains such as attention, motor skills, processing speed, and cognitive flexibility. The instrument was validated for the Brazilian population [50]. In the first part (TMT-A), participants draw a line connecting numbers from 1 to 25 in ascending order. In the second part (TMT-B), participants connect numbers (1–13) and letters (A–L) in an interleaved numerical and alphabetical order. Participants are instructed to maintain pencil-to-paper contact during the test. A shorter completion time indicates better performance.

The COWAT assesses verbal fluency, working memory, and inhibitory control [51]. In this test, participants must say as many words as possible that start with the letters “F,” “A,” and “S” within 1 min for each letter. Proper names, repeated words, and variations in gender, number, and conjugation are not considered. A higher number of words in each test indicate better verbal fluency.

##### Pain

Pain is measured using the BPI, which has been validated for Brazilian cancer patients [52]. This instrument consists of nine multidimensional items that assess pain intensity, pain interference in the patient’s life, pain location, and treatments for pain control and relief. Responses are given on a scale from 0 to 10, reflecting the pain felt at the time of the questionnaire and in the past 24 h. Scores are calculated by averaging the total items. A higher score indicates greater pain severity.

##### Quality of life

The Portuguese version of FACT-B is a valid and reliable tool [53] for measuring the quality of life in breast cancer patients. It is a 37-item instrument designed to measure five domains of quality of life: physical (seven items), social/family (seven items), emotional (six items), functional (seven items) well-being, as well as a breast-cancer subscale (ten items). The responses are presented on a 5-point Likert scale. Participants are instructed to select the number that best represents their response for the past 7 days. Online written permission was obtained to use this questionnaire in the study.

#### Other outcomes

Sociodemographic and clinical characteristics.

Sociodemographic, reproductive, and menstrual characteristics, along with information on family history of breast cancer, oral contraceptive use, hormone replacement therapy, smoking, and alcohol consumption, are collected through a questionnaire before the intervention. Data on tumor histological type, tumor staging, hormone receptor status, and HER-2 expression are obtained from medical records.

##### Physical activity levels

Self-reported physical activity levels are measured before and after 12 weeks of intervention using the Godin-Shephard Leisure-Time Physical Activity Questionnaire. The translated and validated Brazilian version of this questionnaire is used [54]. Participants report the number of times they perform vigorous, moderate, and light physical activities for more than 15 min per week. The weekly frequencies of vigorous, moderate, and light activities are multiplied by nine, five, and three, respectively. The total weekly leisure activity is calculated in arbitrary units by summing the products of each component, allowing for categorization of the individual as insufficiently active or active.

##### Blood pressure outcomes

Office blood pressure measurements are taken at baseline and after 12 weeks of intervention. Participants are kept in a calm environment for 5 min before a researcher measures their blood pressure using a calibrated and automated oscillometric device (Omron Healthcare Inc., Bannockburn, IL, USA). Measurements are initially performed in both arms. Then, three measurements are taken in the arm with the highest value, with a 1- to 2-min interval between each measurement. The average of these three measurements for both systolic and diastolic blood pressure is used.

##### Anthropometric assessment

Anthropometric measurements are taken before and after 12 weeks of intervention. Body mass and height are measured using a digital scale with a stadiometer (Welmy, Santa Bárbara d’Oeste—São Paulo, Brazil). Body mass index (BMI) is calculated using the equation: BMI = body mass (kg) / height^2^ (m). Waist and hip circumferences are measured with a measuring tape, with the tape placed around the navel for waist circumference and around the widest part of the hips for hip circumference. These measurements are used to calculate the waist-hip ratio.

##### Food consumption

The “Food frequency questionnaire as subsidy for programs of non-communicable chronic diseases prevention” was used to monitor changes in food consumption [55]. The instrument is administered at baseline to assess the food consumption frequency over the month prior to the start the intervention and again post-intervention to assess this information during the last month of intervention.

##### Follow-up questionnaire

Each participant completes a questionnaire post-intervention to assess their perceptions regarding safety, enjoyment, motivation, future outlook, benefits for daily life, influence of intervention partners, exercise-related fatigue, satisfaction, self-confidence in physical performance, supervision preference, changes in lifestyle including physical activity and dietary habits, and main barriers to group participation. The questionnaire consists of 14 questions about their individual perceptions of the intervention, using a 7-point Likert scale, in which “1” means “strongly disagree” and “7” means “strongly agree.”

##### Adherence assessments

Adherence is assessed by monitoring both attendance and compliance with the interventions. Attendance is tracked by recording session frequency and is expressed as the percentage of intervention sessions attended by a participant out of the total number of scheduled sessions (24 sessions for exercise programs or 12 sessions for the health education program alone). Compliance is defined as the percentage of intervention sessions performed without deviations from the protocol.

### Plans to promote participant retention and complete follow-up {18b}

To enhance participant retention and ensure complete follow-up, regular and clear communication is maintained to sustain engagement, provide appropriate incentives, and offer ongoing support to minimize dropouts. All participants are also invited to join the ERICA Project after completing post-intervention assessments. Additionally, detailed records are kept of any discontinuation or deviation from intervention protocols, including data from participants in these situations, to ensure a comprehensive and transparent analysis of study results.

### Data management {19}

At the end of each testing day, a responsible researcher verifies missing or inaccurate data and the check the backup data. Subsequently, double data entry is performed for primary, secondary, and additional outcomes. Additionally, ultrasound images of the quadriceps femoris muscles are stored and shared via a secure cloud-based platform (Google Drive).

### Confidentiality {27}

Data are collected on standardized paper forms labeled with each participant’s ID. These paper forms will be stored in LabNeuro for 5 years. After data entry into the computer, the digital data will be stored on a secure drive accessible only by the research team to maintain confidentiality throughout the trial and beyond.

### Plans for collection, laboratory evaluation, and storage of biological specimens for genetic or molecular analysis in this trial/future use {33}

Not applicable. No biological specimens will be collected in the current trial or for future use in ancillary studies.

## Statistical methods

### Statistical methods for primary and secondary outcomes {20a}

We will use descriptive statistics, including mean, standard deviation, 95% confidence intervals, and absolute or relative frequencies as applicable. The Shapiro–Wilk and Levene tests will be used to assess data normality and homogeneity. Generalized estimating equations, with Bonferroni adjustment for post hoc tests, will be used to compare time points and groups. One-way ANOVA and/or Kruskal–Wallis tests will be conducted to compare training compliance and follow-up questionnaire responses between groups. Effect sizes between groups will be calculated based on the absolute difference (± SD) between baseline and post-intervention values, using Cohen’s *d*. All statistics procedures will be performed in the SPSS vs. 28.0, with a significance level set at *α* = 0.05.

### Interim analyses {21b}

No interim analyses are planned. There are no anticipated issues with the intervention that would be detrimental to the participants and require previous interruption of the trial.

### Methods for additional analyses (e.g., subgroup analyses) {20b}

None planned.

### Methods in analysis to handle protocol non-adherence and any statistical methods to handle missing data {20c}

The primary and secondary outcomes will be analyzed according to intention-to-treat (ITT) principles, which include all randomized participants. Missing data will be handled by multiple imputations using generalized estimation equations. Additionally, a per-protocol (PP) analysis will be conducted, including only the participants who completed the trial (completers) with adherence to at least 70% of the intervention sessions.

### Plans to give access to the full protocol, participant-level data, and statistical code {31c}

Access to the full study protocol, participant-level data, and statistical code will be available from the corresponding author upon reasonable request.

## Oversight and monitoring

### Composition of the coordinating center and trial steering committee {5d}

Due to the study’s single-center nature, a specific monitoring committee was not established. However, the two main researchers (CLA or SSP) oversee all aspects of the trial through continuous communication with the study manager (LSA), who coordinates the research team. The study manager is responsible for training the team, providing ongoing support, and taking necessary precautions to avoid unmasking treatment allocation.

### Composition of the data monitoring committee, its role and reporting structure {21a}

The WaterMama Study does not have a data monitoring committee due to limited resources. The Human Research Ethics Committee of ESEF/UFPel does not require this committee, given the low-risk nature of interventions and outcomes.

### Adverse event reporting and harms {22}

Adverse events are systematically collected and classified according to their severity (i.e., mild, moderate, or severe), predictability (i.e., expected or unexpected), and potential relationship to study procedures (i.e., definitely related, possibly related, or unrelated). A multidisciplinary team, including at least two main researchers (CLA or SSP), the study manager (LSA), and medical consultants and experts, discusses all adverse events to determine the appropriate procedures when necessary. Additionally, all adverse events and their classifications will be reported to the Human Research Ethics Committee of ESEF/UFPel. Finally, this data will be fully described in the scientific publications from the trial.

### Frequency and plans for auditing trial conduct {23}

Due to limited resources, the WaterMama Study does not have planned conducting auditing trial. However, the Project Management Group meets at least twice per semester (before and after each wave of intervention) to review the trial conduct.

### Plans for communicating important protocol amendments to relevant parties (e.g., trial participants, ethical committees) {25}

Any necessary amendments to the study protocol will be promptly communicated to the Human Research Ethics Committee of ESEF/UFPel. Concurrently, the main researchers will update the clinical trial registry with the revised protocol.

## Dissemination plans {31a}

Upon completion of the study, our dissemination plan aims to share the findings with as many stakeholders as possible. Participants will receive personalized reports detailing their measurements and interpretations, presented in language accessible to the general public. Additionally, all participants will receive general guidance on breast cancer, overall health care, and physical exercise practice. The study results will also be disseminated to the general public through press releases. For the academic and scientific dissemination, research articles will be submitted to peer-reviewed journals and presented at relevant scientific conferences.

## Discussion

The WaterMama Study is strengthened in the attempt to understand the effects of programs of physical exercise conducted in different environments to expand the knowledge related to water-based exercises for breast cancer survivors, minimizing adverse effects from the disease and its treatment. We have confidence in the potential of water-based exercises to mitigate the treatment side effects, as exercise programs in the aquatic environment may positively impact health-related outcomes [16–19].

Our expectation is that both land- and water-based exercise programs will significantly improve the investigated outcomes compared to the health education group program. Additionally, due to its multi-component nature, we expect the water-based exercise program to positively impact cancer-related fatigue and physical parameters in a greater magnitude. This hypothesis is supported by previous studies demonstrating that aerobic exercise programs in the aquatic environment can increase strength, muscle thickness, and quality in older women due to the multidirectional resistance offered by the water drag force [35, 36].

In addition, it is worth noting that most women breast cancer survivors use hormonal therapy, which comprises among their adverse effects joint pain and other musculoskeletal disorders [33]. The buoyancy of water reduces the apparent weight of adult and older women by approximately 70 to 80%, resulting in a significantly attenuated ground reaction force during water-based exercises [30, 31]. Therefore, this characteristic may make certain types of water-based exercises more feasible for this population than on land, particularly considering the high prevalence of overweight and obesity among breast cancer survivors [56].

Other characteristics inherent to the aquatic environment also seem to improve the range of motion and reduce pain perception in breast cancer survivors [20]. These analgesic effects are caused by the modulation of pain, which is affected by the temperature and turbulence of the water (therapeutic effect of hydromassage) [32]. Consequently, water-based exercise programs may be more pleasant, leading to greater well-being and potentially higher adherence rates throughout life than other exercise programs.

Collectively, the characteristics of the aquatic environment may be beneficial to this population not only in physical outcomes, but also have positive effects on mental health and cognitive function, ultimately enhancing the quality of life for breast cancer survivors. These concerns are relevant because it is estimated that 9.4–66.1% of women diagnosed with breast cancer suffer from symptoms of anxiety and depression, which decreases the patient’s quality of life. Moreover, cognitive problems are common among breast cancer survivors; it is estimated that up to 35% of patients experience cognitive impairments that can last for years after the end of treatments. Therefore, the analysis of such outcomes in water-based exercise programs also deserves attention in the literature [57, 58].

Although previous evidence has evaluated some health-related outcomes in this population following water-based exercise programs [20–29], there is a scarcity of studies comparing the effects of land- versus water-based exercise programs [24, 26]. Finally, we hope that the findings of our study will further strengthen the notion that water-based aerobic exercise is a valuable non-pharmacological tool for recovery and health promotion following primary breast cancer treatment.

## Trial status

This manuscript is based on the trial protocol dated 19 April 2024. At the time of submission, patient recruitment has begun, and it is estimated to be completed up to August 2024*.*

## Data Availability

The data will be available from the corresponding author upon reasonable request.

## Abbreviations

BMI: Body mass index
BPI: Brief Pain Inventory
COWAT: Controlled Oral Word Association Test
ERICA: Exercise Research in Cancer
ESEF: Escola Superior de Educação Física e Fisioterapia
FACT-B: Functional Assessment of Cancer Therapy-Breast
FACT-Cog-v3: Functional Assessment of Cancer Therapy—Cognitive Function—Version 3
HADS: Hospital Anxiety and Depression Scale
HR: Heart rate
ID: Identifying number
LabNeuro: Laboratório de Avaliação Neuromuscular
RF: Rectus femoris
PFS: Piper Fatigue Scale
RPE: Rating of perceived exertion
TMT: Trail Making Test
UFPEL: Universidade Federal de Pelotas
VI: Vastus intermedius
VL: Vastus lateralis
VM: Vastus medialis
VO_2peak_: Peak oxygen uptake
VO_2VT1_: Oxygen uptake in the first ventilatory threshold
VO_2VT2_: Oxygen uptake in the second ventilatory threshold
1RM: One-repetition maximum
